# Perspectives and Experiences of Adult Patients With Obesity in Dietetic Primary Health Care: A Qualitative Study in the Netherlands

**DOI:** 10.1111/jhn.70179

**Published:** 2025-12-15

**Authors:** Annemieke van de Riet, Rebecca S. Otte, Lotte Maree‐Hulsbergen, Elke Naumann, Marian A. E. de van der Schueren, Harriët Jager‐Wittenaar, the Dietetics Building the Future consortium, Mi Sun van der Mannen, Kirsten Berk, Jacqueline Langius, Barbara van der Meij, Martinette Streppel

**Affiliations:** ^1^ Wageningen University and Research, Human Nutrition and Health, Research group Global Nutrition Wageningen the Netherlands; ^2^ HAN University of Applied Sciences, Research Group Nutrition, Dietetics, and Lifestyle Nijmegen the Netherlands; ^3^ Hanze University of Applied Sciences, Research Group Healthy Ageing, Allied Health Care and Nursing Groningen the Netherlands; ^4^ Department of Gastroenterology and Hepatology, Dietetics Radboud university medical center Nijmegen the Netherlands; ^5^ Department of Physiotherapy, Human Physiology and Anatomy, Research Unit Experimental Anatomy Vrije Universiteit Brussel, Faculty of Physical Education and Physiotherapy Brussels Belgium; ^6^ Department of Internal Medicine, Division of Dietetics Erasmus Medical Centre Rotterdam the Netherlands; ^7^ Department of Nutrition and Dietetics and Research Group Rehabilitation and Technology The Hague University of Applied Sciences, Faculty of Health, Nutrition and Sport The Hague the Netherlands; ^8^ Dutch Association of Dietitians Utrecht the Netherlands; ^9^ Department of Nutrition and Dietetics Amsterdam University of Applied Sciences, Faculty of Health, Sport and Physical Activity Amsterdam the Netherlands

**Keywords:** dietetic treatment, nutrition, obesity, patient‐centered care, personalised treatment, weight management

## Abstract

**Background:**

Obesity is defined as a chronic, relapsing disease, characterised by its persistence and the frequent recurrence of weight gain following weight loss. It is linked to chronic diseases and reduced quality of life. Sustainable weight loss requires long‐term dietary and behavioural changes. Dietetic treatment outcomes vary per patient, leading to differing success rates and effectiveness. Although understanding patient experiences is crucial for designing effective, patient‐centered strategies, little is known about their lived experiences. This study aimed to obtain a better understanding of the perspectives and experiences of patients with obesity receiving dietetic treatment in primary health care in the Netherlands.

**Methods:**

This qualitative interpretive study included 26 patients with obesity who were consulting or had consulted a dietitian. We performed purposeful sampling to ensure a sample diverse in socio‐cultural background, region, age and health literacy. Semi‐structured interviews were conducted using a predefined topic guide, with room for flexibility to explore emerging topics. We analysed the interview data using reflexive thematic analysis as described by Braun and Clarke.

**Results:**

Four interconnected themes were constructed from what patients named to be important in dietetic treatment: (1) personalised treatment as a foundation for success, including feeling heard and receiving responsive and adaptive treatment (2) the personal impact of the dietitian on treatment, through the dietitian's attitude, professionalism, and background; (3) a personalised and holistic approach for lasting change, including clarity about the dietitian's ways of working, motivational and emotional support, attention to life context and guidance in developing sustainable habits; and (4) tailored and accessible advice, focusing on practical tools, understandable information, and consistency of advice.

**Conclusion:**

This study highlights that successful dietetic treatment for adults with obesity depends on personalised support, a strong patient–dietitian relationship, and holistic attention to psychological and behavioural factors. Clear communication, involvement from the start, and practical, tailored advice help patients comply with treatment and apply recommendations in daily life. Findings emphasise the importance of aligning dietetic treatment with individual patient needs and expectations.

## Summary

- A strong personal connection, in which patients feel heard and seen, forms the foundation for effective dietetic treatment.
- The dietitian's attitude and communication style strongly shape how treatment is experienced.
- Tailored advice, clear materials, and consistent communication enhance patients' ability to apply dietary guidance in daily life.

## 1 Introduction

Obesity is a complex chronic relapsing disease with globally 650 million people affected [1]. In the Netherlands, 15% of the adult population was classified as having obesity (BMI ≥ 30 kg/m^2^) in 2023 [2]. Patients with obesity have a higher risk of having or developing a variety of chronic diseases, including cardiovascular disease and type 2 diabetes [3, 4, 5]. Moreover, obesity is associated with a lower quality of life and mental health conditions [6, 7, 8, 9]. Weight loss in patients with obesity is associated with significant health improvements, including reduction of osteoarthritis, hypertension, type 2 diabetes, and cardiovascular risk biomarkers [10]. Therefore, long‐term changes in diet and behaviour are required to achieve sustainable weight loss and improved overall health outcomes. Managing obesity typically requires a multidisciplinary or even interprofessional approach, in which dietitians are key in facilitating patients' dietary and behavioural change. Dietetic treatment consists of a holistic interplay of medical considerations, dietary interventions, behavioral counselling, and attention to the patient's social context, all aimed at achieving sustainable weight loss and improving overall health outcomes. Dietitians also help to identify and treat risk factors for malnutrition and nutritional deficiencies [11].

Systematic reviews have shown that weight management interventions by dietitians are efficacious for treating adults with overweight or obesity [12, 13]. However, effectiveness in practice largely differs for each individual. A study using real‐life practice data of patients with overweight in the Netherlands found that only a quarter of patients reached at least 5% weight loss during dietetic treatment. Furthermore, the recommended treatment duration of six months or longer was also only reached by a quarter of patients [14]. Many studies focus on identifying the effect of a dietary intervention within dietetic treatment, rather than understanding why a treatment works or does not work for certain individuals. In these studies, quantitative measures (e.g., weight loss or health intermediates) were predominantly chosen as treatment outcomes [12, 15, 16]. However, understanding patient experiences is crucial for designing effective, patient‐centered (PCC) strategies [17]. Yet, research exploring the lived experiences of patients with obesity remains scarce [18].

Many studies have shown the importance of PCC practices and stated the need for more research into patients' preferences and beliefs to design interventions to better cater to patients' unique needs [19, 20, 21]. Individuals with obesity face specific and multiple challenges, including comorbidities, mental health, and stigma [22], highlighting the need to explore their experiences more in‐depth. Obesity has an unequal distribution across societal groups and is more prevalent among individuals with lower socioeconomic backgrounds [23, 24] and those with limited health literacy, which applies to a quarter of the Dutch population [25]. Literature shows that people with limited health literacy more often struggle to navigate through healthcare. They have difficulty participating in consultations, assessing treatment options and managing health at home [26, 27], calling for an elevated role of dietitians to address health literacy [28]. A low socio‐economic position can hinder patients' capability to afford healthier foods [29] and is in turn associated with limited health literacy [30, 31]. In addition, obesity is more prevalent among people with a migration background, for example, Turkish, Moroccan, Surinam, or Antillean migration background in the Netherlands [32]. Patients' cultural background influences their beliefs about what constitutes healthy food, the role of social environment, and their expectations of health care providers. For minority and ethnic groups across different countries, dietary guidelines may be difficult to reconcile with traditional cooking practices and cultural customs, such as the social importance of hospitality and shared meals. Studies from various contexts have shown that dietary advice may not always align with cultural food preferences and practices, creating additional barriers to adherence [33, 34, 35, 36].

Understanding the factors behind treatment success in individuals with different backgrounds is key. Therefore, the aim of this qualitative interpretive study using reflexive thematic analysis was to understand the perspectives and experiences of adult patients with obesity on dietetic treatment in primary healthcare.

## 2 Methods

The current study is part of the four‐year research project entitled ‘Dietetics building the future’ (Box 1). Findings are reported according to the Standards for Reporting Qualitative Research (SRQR) checklist (See Supporting Information File S1) [37]. Ethical approval was provided by the Research Ethics Committee of Wageningen University & Research (WUR‐REC) under approval number 2023‐034.

Box 1 **Description of research project Dietetics building the future.**

The research project ‘Dietetics, building the future’ is a research collaboration of (applied) universities, practising dietitians and other relevant stakeholders in the Netherlands, to develop an evidence‐based toolbox for personalised dietetic treatment for patients with obesity and comorbidities. The project addresses research questions regarding the optimisation of obesity treatment, improving effectiveness and patients' self‐efficacy. Furthermore, the research project pays special attention to patients with low health literacy, stimulating behavioural change, and the application of innovative technologies. The project consists of quantitative analyses and statistical modeling to identify key components of dietetic treatment. Concurrently, qualitative studies, such as the study on perspectives of dietitians on successful dietetic treatment [38] have been conducted, along with the current study. The results of these studies will be used for the development (co‐design) and testing of a toolbox, offering personalised interventions based on various individual patient factors. A summary of the project can be found on the website of ZonMw, the organisation that funded this research [39].

### 2.1 Participants

This study was carried out throughout the Netherlands. Participants were approached by their primary care dietitian, who invited them to participate in the study. These dietitians were reached via the researchers' networks and various organisations, such as ‘Stichting lezen en schrijven’, the Dutch foundation that focuses on promoting literacy skills. Patients were eligible to participate in the study if they had obesity (BMI ≥ 30 kg/m^2^) at the start of treatment and had consulted a dietitian within the past year for weight loss. Dietitians were asked to inform eligible patients about the study and provide interested patients with information about the study. If patients expressed interest in participating, they either filled out an online registration form via Qualtrics, contacted the researchers directly, or authorised the dietitian to share their contact information with the research team. For registration, patients provided their name, age category, place of residence, and contact details. Interested patients received further study information, and interviews were scheduled at a location of their choice if they agreed to participate. Dietitians were asked to only invite adult patients with obesity (BMI ≥ 30 kg/m²) to participate in the study. However, BMI values were not verified with the participant prior to inclusion.

We aimed to include 25 participants in this study. To estimate the required number of participants, we reviewed similar studies [40, 41, 42], as well as the recommended range in literature for qualitative studies, varying from 5 to 25 [43]. We strived for the upper end of the range, to reach a group of participants with a diversity in demographic characteristics, such as place of residence, socio‐cultural background, and age (see Data Collection—Questionnaire). There were no inclusion criteria regarding the number or frequency of dietetic consultations. Participants were included regardless of whether they were still receiving treatment or had discontinued care, as the study aimed to capture a wide range of experiences, including those of patients with limited and more extensive contact with dietitians. Purposeful sampling was applied to achieve a study sample that is representative of the patient population of primary care dietitians. At the end of each interview, the participant completed a questionnaire to collect demographic information. Throughout the research process, the results of this questionnaire were evaluated to identify gaps in the study population. Special attention was given to recruiting participants with low health literacy, difficulties with reading and writing, and patients with a migration background.

### 2.2 Data Collection

#### 2.2.1 Questionnaire

The questionnaire included the following items: age, gender, postal code, household situation, attained education, work situation, receipt of government allowance, migration background, duration of dietetic treatment, number of visited dietitians during lifetime, reason for referral to dietitian, weight at the start of treatment, current weight, satisfaction with results, literacy, and health literacy. We aimed to include participants from different regions and from both urban and rural areas. The degree of urbanisation was assessed using the urbanisation score from the Dutch Central Bureau of Statistics (CBS), which was calculated based on data for each neighbourhood [44, 45]. Information on education level was presented via the International Standard Classification of Education (ISCED) [46]. Receipt of social allowance was used as a proxy for socio‐economic position, as financial information was considered to be too sensitive for the purpose of this study. The migration background was defined based on the CBS definition, distinguishing between first‐generation (born outside of the Netherlands and at least one of the parents born outside of the Netherlands) and second‐generation (at least one of the parents born outside of the Netherlands) migration backgrounds [47]. The participant's satisfaction with the results of the treatment was assessed using the following question (translated from Dutch): ‘Are you achieving the desired results/the results you had in mind?’. Literacy level was asked with the question, ‘We know that many people find it difficult to read and/or fill out forms. How is that for you?’. Health literacy was assessed via three questions that we translated from screening questions for limited health literacy in a large outpatient population [48].

#### 2.2.2 Topic Guide

A topic guide was constructed based on preliminary results of interviews with dietitians [38], and themes that were identified in scientific literature, related to important components of dietetic treatment [41, 49]. The topic guide consisted of questions related to dietetic treatment, including dietetic guidance, expectations and goals, duration and frequency of consultations, changing diet and habits, communication, support, advice, self‐monitoring, social surroundings, preferences, and expectations for the future. For each topic, several example questions were included in the topic list; examples are provided in Box 2. The supporting information includes the full topic list.

Box 2 **Examples of probing questions for two topics in the topic guide**

**Table:** 

Topic	Probing questions
Expectations & goals	*What were your hopes/expectations for the dietetic treatment?*
	*Did this come true?*
	*Why/why not?*
	*When is the guidance successful for you?*
	*What was/is your goal?*
Treatment & guidance	*Can you tell us something about the guidance from the dietitian?*
	*What did you discuss with the dietitian during the consultations?*

The topic list was tested in the first two interviews with both the first and second authors present to ensure consistency and refine the approach. The interviews were semi‐structured, meaning that the main topics were discussed in all interviews while allowing space for additional topics [50].

#### 2.2.3 Conducting the Interviews

The face‐to‐face interviews took place between December 2022 and January 2024. Interviews were conducted in Dutch by the first two authors, both having a background and expertise in the fields of human nutrition, health sciences and/or dietetics, along with two fourth‐year Nutrition and Dietetics students. These students observed an interview before conducting the interview themselves. Most participants chose to be interviewed at their homes. The informal interview setting made it easier to build rapport, create a comfortable environment for participants, and observe social and non‐verbal cues [51]. One interview was conducted at a neighbourhood centre, upon the request of the participant. The interviews were scheduled with a maximum duration of approximately 60 min, to avoid placing too much burden on participants, while allowing sufficient time for in‐depth discussion of their experiences. Interviews only continued beyond this time when participants indicated they were comfortable doing so.

Participants were informed about the interview aim and procedure via phone, email, and an information letter. They were reminded of the study details and had the opportunity to ask questions before the interview began. All participants provided consent before the start of the interview. This was done in writing or, for those who had difficulty reading and writing, the informed consent was read out loud by the interviewer, and consent was given verbally and audio recorded. At the start of each interview, we built rapport by introducing the study and interviewer, emphasising that the interview was an open conversation and participants could skip any question. After concluding the interview, participants were given the aforementioned questionnaire to fill out. The interviewer assisted with questions that were not clear to participants. For those with reading or writing difficulties, the questionnaire was administered verbally. Participants received a 25‐euro voucher as reimbursement for their time and effort to participate in this study. After completing the interviews and transcripts, summaries of their individual interviews (member checks) were sent to participants, for their review and feedback, which made sure that the representation of their perspectives as written in the summary were correct [52]. Participants could also contact the researchers by phone if they preferred to provide feedback verbally or had questions about the summary. All participants agreed with the summaries. We acknowledge that the written format may have been challenging for some participants with difficulties in reading or writing, which is discussed as a limitation in the Discussion section.

### 2.3 Reflexivity

The background of the research team played a role in this study exploring the experiences of people living with obesity who received dietetic treatment. All authors have an academic background in Nutrition and Health. AvdR, EN, HJ, LMH and MdvdS are registered dietitians and provided expertise on dietetic treatment and professional practice. RO's extensive experience in qualitative research supported depth and reflexivity during data collection.

EN, HJ and MdvdS hold senior research positions and have extensive experience in conducting qualitative research in dietetics, contributing to reflexive discussions about the data and its interpretation. Reflexive discussions were held throughout the research process to reflect on the researchers' assumptions and professional perspectives, and how these may have influenced data interpretation. AvdR and RO were PhD candidates throughout the study and gained additional knowledge about obesity through their involvement in other studies within the project (see Box 1). AvdR, who conducted most of the interviews, had prior experience providing dietetic treatment to people living with obesity and participated in several training sessions on obesity, socio‐economic position, diverse cultural backgrounds, and low (health) literacy, which supported her in interviewing participants with different characteristics. She followed training in qualitative research and had prior experience in conducting interviews.

### 2.4 Data Analysis

The audio recordings from the interviews were transcribed verbatim using Amberscript software. The interviews were analysed following the six‐step approach by Braun and Clarke [53, 54]. This framework guided the entire analytic process, from familiarisation with the data to generating, reviewing, and defining themes, and reflects an interpretive approach in which meaning was co‐constructed between researchers and participants. Following Braun and Clarke's reflexive approach [54], themes were not viewed as passively emerging from the data but were actively developed by the researchers through a process of interpretation and reflexive engagement. Initial codes were generated inductively from the data, and through iterative discussion and review, these codes were grouped and refined into overarching themes that best represented the dataset. First, the transcripts were read by the researchers to become familiar with the data. Second, initial codes were assigned to text fragments, using the program ATLAS.ti Web (version 5.20.0). To ensure consistency, the transcripts of the first three interviews were coded by the first three authors (AvdR, RO, LMH) and during a session, differences in coding were discussed, after which consensus was reached regarding the coding approach. Coding was done inductively, meaning that the researchers gave a code to text fragments without a predefined framework. Nonetheless, codes often aligned with the topics in the topic guide. Third, the codes were reviewed in multiple review sessions by AvdR and LMH and initial themes were constructed. Fourth, themes were discussed by AvdR, LMH, HJ and EN. Fifthly, themes were refined and defined, ensuring that the most important code groups under the themes were represented and subthemes were formulated. In addition, we analysed whether we could see patterns in findings with background characteristics of participants, such as their age or gender. In the sixth and final step, quotations were reviewed to best support the overall findings. These were translated from Dutch to English and reviewed by a colleague native in Dutch and English.

## 3 Results

In total, 26 interviews were conducted. The characteristics of the participants are presented in Table 1. The participants lived across the Netherlands, covering 9 out of the 12 provinces. After including 17 participants, we made extra efforts to recruit an additional 9 participants with reading and writing difficulties and a migration background, as they were not yet sufficiently represented in the study sample. One participant in this study spoke limited Dutch, which affected her ability to elaborate on certain topics, but she was still able to share insights into her experiences and perspectives. The interviews lasted on average 50 min, with a range of approximately 30–70 min. The duration of dietetic treatment varied widely among participants, ranging from 1 month to 10 years. Because of this large variation, the mean duration was 28 months, while the median was 9 months.

**Table 1:** Characteristics study participants.

Characteristics		*N* (%)		*N* (%)		*N* (%)		*N* (%)		*N* (%)
Sex	Female	16 (62%)	Male	10 (38%)						
Age	30–44 years	5 (19%)	45–60 years	15 (58%)	> 60 years	6 (23%)				
Education level^a^	Low	5 (19%)	Medium	9 (35%)	High	11 (42%)	Unknown^b^	1 (4%)		
Urbanisation level of place of residence participant^c^	Very strongly or strongly	19 (73%)	Moderately	5 (19%)	Slightly/none	2 (8%)				
Social welfare allowance	Yes	8 (31%)	No	17 (65%)	Unknown	1^2^ (4%)				
Migration background	Yes, first generation	6 (23%)	No	20 (77%)						
Number of dietitians visited before current dietitian	0	7 (27%)	1	6 (23%)	2–3	7 (27%)	> 3	6 (23%)		
BMI (kg/m^2^) at start of treatment	24–30^d^	2 (8%)	30–35	9 (35%)	35–40	6 (23%)	> 40	6 (23%)	Did not know	3 (12%)
Reason for referral *(multiple answers possible)*	Overweight/obesity	23 (88%)	Type 2 diabetes	10 (38%)	High blood cholesterol	6 (23%)	Hyperten‐sion	2 (8%)	Other^e^	2 (8%)
Achieved desired results	Yes	18 (69%)	No	3 (12%)	Other^f^	5 (19%)				
Literacy	No difficulty	19 (73%)	Sometimes difficulty	4 (15%)	Often difficulty	3 (12%)				
Whether patients indicated to have difficulty with reading and writing										
Health literacy − whether patients asked for help with filling out forms	Never	13 (50%)	Sometimes	10 (38%)	Often	3 (12%)				
− how often patients needed help reading information from the dietitian or other health professional	Never	17 (65%)	Sometimes	5 (19%)	Most of the time	2 (8%)	Always	2 (8%)		
− how often patients had issues reading and understanding forms from the dietitian or other health professional	Never	19 (73%)	Sometimes	4 (15%)	Most of the time	1 (4%)	Always	2 (8%)		

^a^ Education level is presented using the ISCED classification system [46], Low (0–2): basic skills and fundamental education (lower levels, often compulsory), Medium (3–4) secondary education and short‐cycle post‐secondary education. High (5–8): higher education, including vocational and academic programs at the bachelor's, master's, and doctoral levels.

^b^ One participant did not want to share their education level and whether or not they received social welfare allowance(s).

^c^ Urbanisation level is presented using the urbanisation score from the Dutch Central Bureau of Statistics: Score 5 and 6: strongly or very strongly; score 3: moderately; score 1 or 2: none or slightly.

^d^ Although dietitians were asked to include patients with a BMI of ≥ 30 kg/m^2^, in retrospect it turned out that two participants had a BMI below 30 kg/m^2^. They were still included as their experiences were considered relevant to the study aim.

^e^ Other reasons for referral included: lifestyle and prevention; and eating addiction.

^f^ Answers in the ‘other’ category included: ‘not in terms of weight but in terms of health benefit’, ‘not yet’, ‘partly’, ‘still struggling with binge eating’, ‘not yet but taking own responsibility for this’.

**Figure 1:**
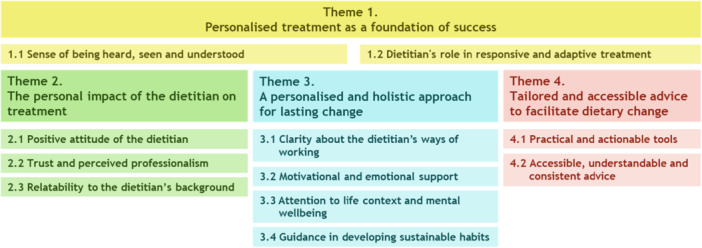
Themes and subthemes.

Participant perspectives on dietetic treatment as discussed in the interviews were captured in four main themes and several subthemes (Figure 1). The themes were described with supporting quotations from participants, including participants' age and gender. When participants had difficulties with reading and writing and/or a migration background, this is also indicated after the quotation.

### 3.1 Theme 1. Personalised Treatment as a Foundation of Success

#### 3.1.1 Sense of Being Heard, Seen and Understood

Participants indicated that a good relationship with their dietitian contributed to feeling supported and more comfortable, and being open and asking questions. According to participants, this facilitated the treatment approach to go beyond nutritional advice by also discussing personal topics and psychological barriers for lifestyle change. Participants identified various factors that contributed to a sense of personal connection, yet they also noted that this connection was often difficult to pinpoint and was more of a feeling than something they could explain.

[A good connection], I do find that important. It's not like I've never really not clicked with someone, but I have the idea I really do with [name dietitian], and whether that's because of her way of guiding or just who she is as a person, that's a bit hard for me to judge, but I do feel like she truly sees me. She sees what I'm struggling with, she sees what it does to me notices how it affects me, and what kinds of problems I run into. I feel like she takes me by the hand and supports me.

(Female, 54 years of age)

Participants sometimes mentioned dietitians' background characteristics (Theme 2.3) when they talked about whether or not they felt a connection, such as the dietitian's age or weight. Factors that foster this connection included feeling seen and heard, the dietitian having an eye for the patient's everyday life and struggles, and offering support in these areas. A lack of personal connection was mentioned by participants as a reason to stop seeing the dietitian.

#### 3.1.2 Dietitian's Role in Responsive and Adaptive Treatment

Participants stressed the importance of dietitians aligning their treatment approach and advice with the patient's individual needs. Discussing these preferences, needs, experiences and skills and knowledge levels at the start of treatment was mentioned as being important for feeling heard, getting the right ‘diagnosis’ and receiving tailored treatment.

That's just something a dietitian should check in advance, in one of the first conversations. That you just ask: so, what do you prefer? Do you like it when I'm really strict, or a bit more relaxed, or what? With both (dietitians) I didn't really have an introduction or intake conversation (…) not like: okay, what would you prefer? What should we be working towards, or anything like that.

(Male, 53 years of age)

Participants pointed out that they often know their own pitfalls but need guidance and support on how to overcome them.

[Interviewer: what is important for dietitians to improve obesity treatment and make it better suited to patients?] Maybe something like, you know, a learning style test like Kolb's, [explanation: Kolb's test categorises four types of learners: diverging, assimilating, converging, and accommodating]. And then have a look at what category someone more or less falls into, and what kind of treatment or support would be appropriate. Because well, I've been at this for four years now, and have since come to know my pitfalls. But actually making changes, that still isn't working, and that's a shame.

(Female, 34 years of age)

Participants mentioned that they did not always share all relevant personal circumstances with the dietitian at the beginning, such as challenges with reading and writing. According to participants, it is important for the dietitian to explore needs that might be sensitive in a subtle manner. Participants noted that limited finances can hinder initial or continued visits to the dietitian beyond insured consultation hours and they appreciated the dietitian taking this into account.

You can ask it in the first conversation: how do you fill things in, or can you read things? (…) But you have to ask it in the right way, not immediately say: ‘Do you have difficulties with reading and writing?’ But more like: how do you read the products? Do you understand the products, do you know what the products are, because then you understand the calories.

(Male, 52 years of age, difficulty with reading and writing)

Participants expected the dietitian to be able to change the treatment approach in case of changing needs or when progress stagnated. Changing the approach was considered necessary when the same issue was discussed repeatedly without new insights or results, according to participants. They reported not always knowing what the dietitian could do differently as they lacked information on treatment options.

Participants described how feeling heard, seen, and understood was essential for engaging with other elements of the treatment process. This initial sense of recognition enabled them to build trust in the dietitian (Theme 2), feel supported in a personalised and holistic way (Theme 3), and make use of tailored advice in daily life (Theme 4). When this foundation was lacking, the overall treatment experience was perceived as less positive and supportive.

### 3.2 Theme 2. The Personal Impact of the Dietitian on Treatment

#### 3.2.1 Positive Attitude of the Dietitian

Participants appreciated a positive attitude from the dietitian, highlighting qualities such as empathy, respect, humour and an open, non‐judgmental approach. The feeling of being heard and listened to was described as essential for feeling treated as a unique individual.

She did manage to motivate me more [than the other dietitian]. (…) Maybe also because she was so enthusiastic. If I had lost weight, she'd say: wow, how did you do that? Did you walk more, or did you, what did you do, because it's going so well? That kind of thing.

(Female, 68 years of age)

Furthermore, a curious attitude was much appreciated by participants as a sign of genuine interest from the dietitian. Participants shared that they disliked the dietitian's attitude when it felt impersonal, shallow, or when the dietitian was lecturing in a negative manner.

That was something I missed with my previous dietitian, it felt a bit clinical: you go there, you tell your story, and of course in the beginning that stays quite superficial. And if no further questions are asked, and I'm also not the kind of person who just lays everything out there, then you don't get to that deeper level. And that's exactly what I needed to be able to take those first steps in changing my lifestyle.

(Female, 52 years of age)

Participants shared examples of how past experiences with dietitians shaped their perception of the dietitian. Negative encounters were said to contribute to resistance and distrust towards dietitians, according to participants. These experiences were sometimes intertwined with broader societal stigma around weight and health.

Back then, you went to the general practitioner and they referred you to a dietitian, and I never found them friendly. Not at all, ‘no, you have to do this, you're not allowed that’. That was the mentality back then, and it really created resistance in me. (…) I quit, I just started eating again (…) Well, that lasted a long time, because I developed such a dislike of dietitians. Because of how they approached the diet and how they treated me, and of course also the outside world.

(Female, 55 years of age)

#### 3.2.2 Perceived Professionalism and Trust

Participants expressed appreciation for the professionalism and expertise of dietitians and linked this to their trust in the dietitian's treatment approach. They felt that it was not essential for dietitians to know everything immediately but valued the dietitian's initiative in seeking answers and information. Participants had mixed views on the dietitian's age; some associated a young age with lower perceived expertise, and some were surprised by the quality of advice provided by a young dietitian.

I do notice that she [current dietitian] is really driven, very professional, because I have seen [a dietitian] before, two years ago (…) and that girl just didn't have that much experience yet. (…) When I got home, I called and said, well, I'm not coming back, because (…) yeah, yeah, putting together a quick menu, I can also do that myself.

(Male, 52 years of age)

Some participants linked trust in the dietitian's expertise to their weight, feeling that being too thin or overweight affected their ability to relate and trust the dietitian regarding advice on weight loss. Participants also mentioned that the appearance and equipment of the dietetic consultation room also influenced their impression of the dietitian. A visually informative consultation room contributed to the sense of the dietitian's expertise, contrasting with less engaging, sterile spaces, according to participants. They expressed frustration when essential equipment in the consultation room was repeatedly unavailable.

One of the dietitians had her own room in a health center, and it was full of food examples and posters about nutrition topics, and well, it was like walking into some kind of food heaven. That's the feeling I got, it somehow gave an impression of expertise. That was quite different from when I saw another dietitian who was located in a very sterile room within the care hotel where I was staying. I don't know, but apparently a room like that also makes an impression.

(Female, 44 years of age, migration background)

#### 3.2.3 Relatability to Dietitian's Background

Participants had varying perspectives on the relevance of a dietitian's (cultural) background. For individuals with a migration background, a shared cultural background was valued by some for reducing language barriers and a shared understanding of specific dietary customs or treatment style. Others reported that they found the dietitian's cultural background less relevant because they adhered mainly to a Dutch eating pattern.

You don't have that in Turkey, when you see a dietitian, that they don't perform any blood tests before starting. I find that quite strange, because a lot of things could be related to that.

(Female, 36 years of age, migration background)

I think that if my father were to get advice from a Turkish dietitian, for example, he might be more likely to accept it, because a Turkish dietitian might have a better understanding, knowing the dietary pattern. (…) But for me it wouldn't matter what the dietitian's background is. (…) My son and I also consume Dutch foods, just a sandwich, meat, vegetables, you know.

(Female, 47 years of age, migration background)

Other characteristics mentioned in the interviews included whether the dietitian was extravert and the dietitian's age that made the patient feel like the treatment would work or not. Furthermore, the dietitian's weight was mentioned as something that participants could or could not relate to.

I noticed that that [the personal connection] was actually quite important. I didn't really have that with her. And she was also super slim. So going there wanting to lose weight, you think: ‘why are you so slim?’ You know, that doesn't feel fair (laughs). (..) I joked to friends that she was 21 and could fit through a keyhole, you know, that was how it felt for me. I just did not really have a click with her.

(Female, 50 years of age)

### 3.3 Theme 3. A Personalised and Holistic Approach for Lasting Change

#### 3.3.1 Clarity About the Dietitian's Ways of Working

Participants who lacked a clear idea about treatment approaches expressed the need for information on how dietitians work, in order to help them choose a dietitian and an approach that best fit their needs.

Yeah, and most dietitians do have information about: this is my education, I'm this age, and I live here or something like that, but a lot less about what their guidance style is. Or like, what their highlights are. That way, you could be much more targeted, and it would save you from having to meet with a lot of different people first.

(Female, 34 years of age)

A clear understanding of the dietitian's ways of working and expertise was reported to enhance the connection between patient needs and the treatment approach, making it easier for patients to able to express their needs related to treatment focus.

There just had to be a match with where I was at, you know? And I've been lucky with that, but also just by pushing and asking and saying: No, I don't want it like that, because otherwise I'll stop seeing this dietitian too, because for me, it would just be another story from the Middle Ages.

(Female, 58 years of age)

Several treatment approaches were discussed, among which Acceptance and Commitment Therapy (ACT) was mentioned multiple times as a treatment approach that can help with exploring underlying psychological mechanisms of behaviour. Participants who received dietetic treatment with more emphasis on psychological support and counselling often described this as having a particularly meaningful impact on their experience of treatment.

I've seen other dietitians in the past, where it was very much focused on nutrition and keeping track of lists (…). But I was really looking for someone who could support me with the mental aspect of it. Because every time I struggle, when I'm not feeling good or something emotionally intense happens, I experience setbacks with the dietary pattern. So I was really looking for someone who didn't just focus on lists and diets, but really looked at what's behind it all.

(Female, 54 years of age)

However, other participants shared that they felt hesitant to discuss matters with a psychological focus. They expressed discomfort with exposing personal aspects of themselves or felt that this was not the role of a dietitian, or shared examples of having to do exercises that just did not match their style.

#### 3.3.2 Motivational and Emotional Support

Participants valued the different roles a dietitian can fulfill, particularly emphasising the role of an external motivator. This role was described by participants in various ways: merely being there once in a while to check in but also offering support and encouragement. When asked whether another person or health professional could fulfill this role, participants reacted negatively, expressing that they preferred their dietitian to take on this supporting role.

Well, a lot of people still say: ‘Yeah, you don't really need a dietitian, actually.’ And yeah, maybe that's true. But for me, I mostly did it to have someone to hold me accountable.

(Male, 57 years of age)

Another role of the dietitian that was mentioned was to hold up a mirror and support participants become aware of their behaviour.

And she did mention to me, like: maybe you should start moving more. But for me, that's not the way to go. (…) No, I don't want to be told what to do, and then I'm quick to dig my heels in. Yet somehow, she's really good at seeing through that. And she said: ‘Hey, I see you've got a smartwatch’. Then I thought, oh yeah, I could actually use that, and since then, I've really been keeping track of things: cycling a bit, or when at the gym, on the treadmill or the cross trainer.

(Female, 52 years of age)

#### 3.3.3 Attention to Life Context and Mental Wellbeing

Participants shared their need for dietitians to take a holistic approach by paying attention to a wide range of personal factors that are related to living with obesity rather than focusing only on diet.

When someone is often stressed, you can give them all kinds of nice little things. But first you have to deal with the stress. (…) Not just at the dietitian's, but already with your general practitioner, because they are the one referring you. But why do you have stress? It could be debts, it could be other things, it could be something in your private life. That's something a doctor should already know, so they can record it in the paperwork: you have stress because of this and that, and that should be looked into.

(Male, 52 years of age, sometimes difficulty with reading and writing)

Participants emphasised the importance of discussing mental wellbeing and the underlying causes of eating and lifestyle behaviours with their dietitian, noting that this was sometimes lacking in their treatment. Furthermore, they reported barriers for weight loss included self‐image and confidence to go out in society and to the gym or swimming pool for example. Learning how to view oneself with kindness was mentioned as an important element in improving mental state and confidence.

Well, when you're overweight, you feel unhappy, and you start to exclude yourself from society. You stop going to things, and because of that, you end up moving even less. Or if you feel unhappy, you might actually start eating more. That really is a pattern.

(Female, 58 years)

To facilitate these subjects, they mentioned the possibility of having different health professionals involved, maintaining communication between each other, and ensuring continuation of treatment across different health services. Participants gave examples such as the connection with the general practitioners for identifying lifestyle issues, as well as involvement of a psychologist or mental health worker in the general practice for support with stress and mental health.

I think she [current dietitian] had already been somewhat informed by the [other] dietitian at *institution* about what we were doing back then, and that we've now been able to more or less continue from there, and I really appreciate that.

(Male, 41 years of age)

When it came to food, I could work on that in a practical way with the dietitian. And with the mental health worker I was able to give space to the mental side of things.

(Female, 52 years of age)

#### 3.3.4 Guidance in Developing Sustainable Habits

Participants stressed that lifestyle change is difficult and gave examples of various facilitating and hindering factors for behavioural change. They stressed that their motivation was an important facilitator in their lifestyle change. The dietitian's support was reported to be valuable to stay motivated in the process. Participants shared that the dietitian had helped them to discover their true motivation, and how important this was for them to persevere in their lifestyle change trajectory.

[Motivation is] necessary if you want to achieve something, and it's not easy: changing behaviour and losing weight. So I think motivation deserves more attention. Because I went there to lose weight, that was my motivation, but actually, it was about my health and my hip, and I never really realised that. If I had had that clearer in mind, I might have been able to stick with it longer. (…) I think it's very easy to get bogged down in nutritional advice and the details, and to lose sight of the bigger picture, the motivation, along the way.

(Female, 47 years of age, migration background)

Getting more insight into their own behavioural patterns was mentioned by participants to be an important aspect of dietetic guidance. In addition, participants valued their dietitian's guidance in dealing with setbacks.

It's mainly about becoming aware (…): like what's happening, why are you putting that in your mouth? Did something happen beforehand, what does it do to you? And just through Acceptance and Commitment Therapy and [name dietitian], we look at it like: okay, that happened, you can't undo it, but what did go well? (…) That awareness really gives me a lot of support.

(Female, 54 years of age)

Participants recognised the importance of adopting an eating pattern designed for the long term to help prevent weight cycling. Participants highlighted that the dietitian's integration of social context into their guidance, was supportive in their treatment process.

You can start counting calories and lose some weight in a short time, but when you go back to eating ‘normally’, quotation marks, it tends to comeback quickly. So I really had the feeling I wanted to do something that would work in the longer‐term.

(Female, 50 years of age)

### 3.4 Theme 4. Receiving Tailored and Accessible Advice to Facilitate Dietary Change

#### 3.4.1 Practical and Actionable Tools

A wide range of tools that dietitians used were evaluated by participants as helpful in changing their diet. These tools included example menus, variation lists, recipes, food swaps, and general information about healthy eating. Participants listed methods to reduce portion size, such as how to differently divide food over the plate and smaller portions by measuring. In general, being aware of current eating habits through eating diaries and evaluating this with the dietitian was considered to be insightful for becoming aware of unhealthy patterns.

What I especially liked was that list, actually. I have to say that in the end, that list turned out to be my biggest tool. In that conversation, we also went through my daily menu, like, what do you eat in a day and what kind of food switches can you make?

(Female, 50 years of age)

Different needs were described by participants regarding the content of the advice, such as the level of detail and length of the advice. The dietitian helping them to take small steps was pointed out as an important facilitator for staying motivated and achieving goals.

And small steps, and that's what actually made it very manageable, very easy because of those small steps. You reached your goals easily. That really motivated me, you know? Serotonin is released, we keep going!

(Female, 52 years of age)

Participants again stressed that it is crucial that the dietitian's advice aligns with their current eating pattern and culture. Not being sensitive to personal eating culture and having to adopt a new eating pattern too different from the current pattern was often described as a dealbreaker for participants and a reason to discontinue treatment.

At the dietitian back in Turkey that year, I actually got a list with exact times, like in the morning I had to eat this, in the afternoon that, but here in the Netherlands I didn't get that. (…) Having fixed times does help with structure, which I like, but of course it also takes a lot of preparation to eat those specific things. So I'm a bit torn about it, I think.

(Female, 36 years of age, migration background)

Apart from advice related to dietary changes, examples were given of advices on how to deal with the food environment, such as where to find healthy products in the supermarket.

I used to buy my stuff in the middle aisles of the supermarket. But that's where the soups, the chips, the cookies are, all the bad stuff. (…) I'm a bit angry at the food industry for being allowed to do that, that they get the chance in this free country to do it. (…) I was at an airport last week and I thought: what if I wanted to eat healthily here? How would I even do that?

(Male, 67 years of age)

#### 3.4.2 Accessible, Understandable and Consistent Advice

Participants appreciated different forms of advice such as information on paper, in books and social media accounts with nutrition information, and the use of pictures. The alignment of materials to individual needs was regarded crucial. They emphasised the importance of ensuring that the content was easy to understand, concise, and visually supported with images and pictures, especially for those who struggle with reading and writing. While apps were considered effective for digitally literate individuals, others found them difficult to navigate. Apart from content and length, the design of materials was mentioned as well as being important.

Speaking is difficult for me. With support from my son or daughter, it's okay. But in the past, they always gave a book with pictures, for bread, for food. What I should eat. How much, 100 grams, 150? That was before, [at the dietitian in the hospital], but not anymore. (…) The dietitian is all good, I just want pictures. The pictures are important for me, because I cannot read.

(Female, 66 years of age, migration background, difficulty with reading and writing)

The material that is given to patients to fill out can also be used by the dietitian to assess whether the form and level is appropriate, as pointed out by one participant:

Then they're told to keep a diary, and yeah, people don't know how to write it down properly, so what do you get? They just keep writing the same things over and over. I always tell everyone: take that booklet, go through it carefully. Because that's how you also pick up on people from other cultures or people who have difficulties. A lot of people won't say that they have low literacy.

(Male, 54 years of age, sometimes difficulty with reading and writing)

Participants stressed that inconsistent advice was confusing for them. Examples were provided of both inconsistent advice from one dietitian, and no continuity of advice between different dietitians.

What I find difficult is that I've had different dietitians now, and the one from about 6 months ago said: [Product X], well, that's almost the devil, because it contains sugar. And then another one says: no, [Product X] is a perfectly fine option. And then at some point, you just don't really know anymore.

(Female, 34 years of age)

## 4 Discussion

The aim of this interpretative qualitative study was to understand perspectives and experiences of adult patients with obesity on dietetic treatment in primary healthcare. We found that patients experience personalised treatment as a foundation for successful dietetic treatment (Theme 1). Feeling heard, seen and understood, and receiving responsive and adaptive support, were described as essential to building trust and engaging with the treatment process. This experience was closely connected to how patients perceived the dietitian as shaping their overall treatment experience (Theme 2), including the importance of the dietitian's attitude, professionalism, and background. Patients also valued a personalised and holistic approach that supported lasting change (Theme 3) and described tailored and accessible advice as crucial to putting treatment into practice in daily life (Theme 4). These four themes are interrelated and together shape how participants experienced and evaluated dietetic treatment.

Patients considered strong alignment with their personal circumstances, needs and wishes to be crucial for good dietetic treatment. Feeling heard and a strong connection with the dietitian were central to building trust and engagement. This aligns with previous research highlighting the importance of the therapeutic relationship, communication and provider approach as core components of patient‐centred care (PCC) [55]. A review of therapeutic alliance in obesity care showed that few interventions integrate such elements, underlining the need for more attention to this in dietetic practice [56]. In our study, the quality of the connection was shaped by perceived expertise, advice, treatment style, and even characteristics such as the dietitian's weight, age and experience. This reflects what others have described as the perceived ability of the professional to assist [57]. Patients valued the dietitian as an external motivator and counsellor, rather than just an information provider, a view also expressed by dietitians in our previous study [38]. Although patients appreciated a counselling role, their preferences varied regarding the extent to which psychological topics should be addressed by the dietitian. Other studies have noted that while some patients prefer information over counselling, a purely informative approach can hinder continued engagement in treatment [58, 59]. Patients also reported limited awareness of available treatment options or different working styles among dietitians, reflecting low levels of shared decision‐making, a pattern confirmed in earlier research [60, 61]. Introducing shared decision‐making early in the process may strengthen collaboration and improve the therapeutic relationship [62]. Information provision and attentive listening are key determinants of how patients perceive shared decision‐making in the management of chronic illnesses [63]. Shared‐decision making in turn is an important component of PCC [21] and is linked to patients' self‐efficacy, behaviour change [64, 65, 66] and improving patients' understanding and confidence in managing chronic diseases [67]. This supports a broader shift in dietetic care from prescriptive advice to a more collaborative approach [68].

Our study indicates that patients value the dietitian's ability to understand and respond to their individual reality and needs. A clear need for tailored treatment emerged from the interviews, independent of patient background. However, several points of attention were raised for specific patient needs. These include the need of ensuring the accessibility of information for individuals with literacy challenges and tailoring interventions to align with cultural contexts. This is consistent with other studies on patient needs among ethnic minorities in the Netherlands, in which respondents expected a more technical medical approach to dietetic care [33] and an approach reflecting the healthcare culture in their country of origin [69]. In addition to a tailored approach, patients expressed their appreciation of advice and information material, which needed to be clear, practical and consistent, aligning with previous research [41, 70].

Many findings from our study apply to a broad group of patients, but there are key considerations specifically for patients living with obesity. Patients valued dietitians who went beyond providing dietary advice, instead addressing broader lifestyle and psychological factors while integrating dietetic treatment into overall healthcare. These findings are supported by literature indicating that patients with obesity require holistic long‐term support focused on both diet and a therapeutic counselling approach, integrating behavioural and lifestyle guidance [59, 71, 72]. The need for attention for psychological factors found in our study can be explained by various psychological challenges that particularly are present in patients with obesity, such as dealing with stigma [73], and using food as a coping mechanism [74], which has been related to weight gain or loss [75]. Patients in our study who received dietetic care with a stronger focus on psychology and counseling shared that this had an important impact on their experience of care. Studies have indeed noted the need to focus on self‐acceptance and intrinsic motivation [76] to address the effects of stigma [74, 77] in treatment of patients living with obesity. Although many aspects valued by patients reflect current best practice principles, our findings suggest that these are not always realised in daily practice. Patients pointed to factors such as limited consultation time, variation in counselling approaches, and differences in communication style as possible explanations for this gap.

### 4.1 Strengths and Limitations

This study is the first qualitative study in the Netherlands, and one of the few internationally, that explored the perspectives of patients with obesity on dietetic treatment in primary care, offering unique insights into their experiences. The inclusion of participants with diverse backgrounds strengthened the study by capturing a broad range of perspectives. Despite differences in how dietetic treatment is organised across countries, the insights from this study are highly relevant to dietetic practice and offer valuable perspectives for broader healthcare contexts. As with all qualitative research, the interpretation of the data was influenced by the backgrounds and perspectives of the research team. The researchers' experience in dietetics and qualitative research contributed to the depth and contextual understanding of the findings, while reflexive discussions throughout the study helped to minimise potential bias and strengthen the credibility of the analysis. The study included only a limited number of patients who discontinued care early, which restricts our understanding of their experiences. Another limitation was that the summaries of individual interviews (member checks) were sent in writing, which might have impacted the reviewing ability of participants had difficulty with reading and writing.

### 4.2 Practical Implications and Future Research

This findings of this study highlight the need for a strong emphasis in dietetic treatment on the relationship between dietitian and patient, to significantly enhance treatment experience. Additionally, dietetic treatment for patients with obesity should prioritise personalised sustained support and long‐term behavioural change, with a focus on addressing the psychological aspects that are essential for managing obesity as a chronic condition. Future research should focus on investigating the diverse needs of patient subgroups to more thoroughly understand their unique perspectives, including factors such as health literacy, socio‐cultural and economic background, as well as patients' motivation and self‐efficacy. In addition, future studies could develop and evaluate specific tools that support delivering a personalised treatment. As part of the Dietetics Building the Future research project, a first step has been taken through the development of a toolbox designed to help dietitians address diverse patient needs in daily practice.

## 5 Conclusion

The aim of this interpretative qualitative study was to understand perspectives and experiences of adult patients with obesity on dietetic care in primary healthcare. This study shows that effective dietetic treatment for adults with obesity in primary healthcare is grounded in personalised support and a strong therapeutic relationship. A sense of being acknowledged and understood fosters trust and engagement. The dietitian's attitude, professionalism, background and ability to connect play a central role in how patients experience treatment. A holistic approach that incorporates psychological factors contributes to sustainable lifestyle changes. Practical, tailored advice enhances patients' ability to integrate treatment into their daily lives. These findings underscore the value of responsive, personalised treatment, starting with clear information and involvement even before treatment begins. Future research should focus on strengthening communication and developing different strategies that align treatment with each patient's needs and expectations.

## Author Contributions

**Annemieke van de Riet:** conceptualisation, methodology, data collection and analysis, writing – original draft, review and editing and project administration. **Rebecca S. Otte:** conceptualisation, methodology, data collection, project administration, critical review of manuscript. **Lotte Maree‐Hulsbergen:** data analysis, critical review of manuscript. **Elke Naumann:** conceptualisation, methodology, supervision and critical review of manuscript. **Marian A. E. de van der Schueren:** conceptualisation, methodology and critical review of manuscript. **Harriët Jager‐Wittenaar:** conceptualisation, methodology, supervision and critical review of manuscript. All authors read and approved the final manuscript.

## Ethics Statement

Ethical approval was provided by the Research Ethics Committee of Wageningen University and Research (WUR‐REC) under approval number 2023–034.

## Conflicts of Interest

The authors declare no conflicts of interest.

## Supporting information

Supplementary file_SRQR checklist_22Nov25.

Supplementary material_Topic guide.
