# Supplementary material for: Perspectives and Experiences of Adult Patients With Obesity in Dietetic Primary Health Care: A Qualitative Study in the Netherlands
Source: J Hum Nutr Diet. 2025 Dec 15;38(6):e70179. doi: 10.1111/jhn.70179 (PMC12706137; doi:10.1111/jhn.70179)
Supplement: Supplementary file 2 — Supplementary material_Topic guide. [file JHN-38-0-s001.docx]

**Topic guide**

***Before starting the interview***

**Introduction:**

- I am…, I work at….
- Thank you for your effort and time

**Explanation of the background and purpose of the study:**

- *Dietetics Building the Future* is a research project conducted by several universities of applied sciences in collaboration with Wageningen University.
- In this study, we are interested in your experiences with your dietitian. The interviews will help us understand what works well in dietetic treatment and what aspects could be improved.

**Information about the interview:**

- The interview will take approximately 45 minutes to one hour.
- There are no right or wrong answers; we are simply interested in your personal experiences.
- You do not have to answer any questions you would prefer not to. You may interrupt me at any time, ask for clarification, or stop the interview without giving a reason.
- It is also perfectly fine if the conversation drifts off topic.
- Regarding data handling and the audio recording: all information will be pseudonymised (made anonymous) and treated confidentially. Nothing you say will be shared with your dietitian.

| Topics | Example questions |
| --- | --- |
| Main topic: What factors influence whether or not the treatment is successful? | |
| Introduction | - What made you decide to take part in this interview? - What was the reason you made an appointment with the dietitian? - How did you find or get referred to this dietitian? - Have you ever been to a dietitian before? - If yes: was it the same dietitian or a different one? Could you tell me a bit more about that experience? Did you stop seeing that dietitian (and if so, why)? |
| Treatment/ guidance  Expectations and goals | - What were your hopes or expectations for the treatment? Have these been met? Why or why not? What was your personal goal? - When would you consider the treatment or guidance to be successful? - Could you tell me something about the guidance or support you received from your dietitian? - What did you and your dietitian discuss during the consultations? - Did the dietitian regularly discuss the progress and results of your treatment/guidance with you? |
| Duration, frequency, etc. of the treatment | - How often do/did you see your dietitian? (Was this your own choice, or initiated by the dietitian, for example, because of the three hours of reimbursed care?) - What do you think about this amount/frequency of contact? - Is there any contact outside the consultations? - In an ideal situation, what would the contact with your dietitian look like? |
| Change | - Have you changed any of your behaviours or habits during or after the treatment? - What helped you to make that change? - What was the role of the dietitian in the changes you made? What could the dietitian do to support you in making changes? - What made it difficult for you to change? |
| Dietitian | - What did you expect from your dietitian (what role did he or she have)? - In what way do you (did you) appreciate the dietitian helping or motivating you to make changes? (follow up if needed) - Have your expectations been met? - What is/was your relationship with your dietitian like? (Did you feel a good connection or “click”?) - What do you consider important in your relationship with your dietitian? |
| Communication by the dietitian | - Could you tell me a bit more about the way the dietitian communicated with you during the consultations? - Did the dietitian ask you a lot of questions? - Who did most of the talking during the consultations, you or the dietitian? And did you feel comfortable with that balance? |
| Providing feedback | - Did the dietitian review with you whether you were on the right track, what was going well and what could still be improved or changed? - Did you find that helpful or not, and why? |
| Support | - How important is support from the dietitian for you in achieving your goals? - Did you feel supported by the dietitian during the process? - What gave you that feeling, or what made you feel that support was missing? |
| Content-related advice | - What kind of information (advice, leaflets, or other materials) did you receive from the dietitian during your treatment? - In what way did you receive this advice? - What did you think of this advice?   *Follow up questions:*   - Were you able to apply the advice yourself? Did you feel that the dietitian’s advice was helpful to you? - Which advice was easy to follow, and which was more difficult, and why? - Did the dietitian give advice verbally, or also use other means such as leaflets, websites, or apps? |
| - If “diet” or dietary changes come up here, ask follow-up questions. | - What kind of advice did the dietitian give you about your diet or eating pattern? - Was this advice clear to you? - How was it for you to follow this advice? Have you been able to maintain it? |
| Self-monitoring | - Do you monitor your own weight or health? How do you do that? - Did the dietitian ask you to do anything at home to monitor your health? (For example, weighing yourself or using a pedometer.) |
| Social environment | - What role did your partner, family, or friends play during your treatment with the dietitian? - Did they play a role in the changes you did or did not make? - Did the dietitian pay attention to the role of your family or friends? (follow up if relevant) |
| Preferences | - What aspects of the dietitian’s guidance helped you the most in achieving your goals or during the treatment? - What did you find difficult or less pleasant during the process? - Was there anything you felt was missing during your treatment with the dietitian? - Are there things that could have helped you more in your treatment? Or things you would not have wanted to miss — that were very important to you? |
| Future | - Have you been able to maintain the changes after finishing your treatment with the dietitian? - Do you think you will be able to maintain the changes once you stop seeing the dietitian? - …and why or why not? What do you think you would need to keep it up? - Would you recommend seeing a dietitian to family or friends who are in a similar situation as you? |
| Other topics | - Is there anything else you would like to share about your experience with the dietitian? |

**End of the interview**

- We’re coming to the end of this interview. Is there anything else you would like to share about your experience with the dietitian?

**Thanking the participant**

- Thank you very much for your time and effort.
- Hand over the gift voucher.
- Explain that participants will receive a short summary of their interview to check whether it reflects their experiences accurately.
